# Selective Blocking Property of Microporous Polymer Membranes Fabricated by Chemical Vapor Deposition

**DOI:** 10.1038/s41598-017-15470-x

**Published:** 2017-11-15

**Authors:** Takeshi Shii, Masaru Hatori, Kazuma Yokota, Yoshiyuki Hattori, Mutsumi Kimura

**Affiliations:** 1 Department of Chemistry and Materials, Faculty of Textile Science and Technology, Shinshu University, Ueda, 386-8567 Japan; 2 Global Aqua Innovation Center, Shinshu University, Nagano, 380-8553 Japan

## Abstract

Poly-*p*-xylylene films have been utilized as protective and barrier layers for gases and solvents on electronic and implantable devices. Here we report a new approach to create highly permeable and selective nanofiltration membranes coated with microporous poly-*p*-xylylene nanofilms fabricated through a dry chemical vapor deposition process by using [2.2]paracyclophanes derivatives on ultrafiltration membranes. The introduction of crosslinking points into rigid poly-*p*-xylylenes enhanced microporosity and mechanical strength due to insufficient packing and depression of structural relaxation among polymer chains in three-dimensional networks. Crosslinked nanofilms with thicknesses down to 50 nm showed outstanding permeability for water and alcohols at a pressure difference of 0.5 MPa and exhibited higher rejection ratios for water-soluble organic dyes than non-crosslinked nanofilms. Poly-*p*-xylylene nanofilms also showed an excellent blocking property for non-polar organic solvent permeation through specific interaction of hydrophilic pores with organic solvents.

## Introduction

Membrane separation process has been widely used in various application fields including water purification, gas separation, and processing in the pharmaceutical and food industries owing to its high-energy efficiency^1–4^. To enhance its performance, high permeability and selectivity are required. Conventional polymer separation materials have a performance trade-off between the permeability and the selectivity. The target small molecules can penetrate selectively by passing through pores in the dense packing of rigid polymer chains. However, the dense packing of rigid polymers results in a poor permeability. To overcome this trade-off, the polymer has been designed at the molecular level, and the separation layer has been made thinner to provide sufficient permeability and high selectivity for membranes^5,6^.

Microporous organic polymers (MOPs) are a notable candidate for highly permeable and selective membranes^7–9^. The inefficient packing among contorted and rigid MOP chains creates intrinsic voids of less than 2 nm diameter, and thin MOP layers provide both the permeability and selectivity to support scalable separation processes^10–15^. Recently, Livingston *et al*. succeeded in fabricating defect-free crosslinked-polyester nanofilms on porous supports made by the interfacial polymerization^16^. The crosslinked nanofilms with controlled microporosity showed a much better separation performance in organic solvents than films made from non-contorted planar monomers. The crosslinking of contorted polymers in the nanofilms enabled the permeability of organic solvents to be enhanced while maintaining the membrane selectivity. Although the interfacial polymerization has been used as a simple and controllable technique to prepare the crosslinked polymer films in reverse osmosis desalination and nanofiltration (NF)^1–4^, the ester or amide linkages in these condensation polymers can be broken by hydrolysis and oxidation. Therefore, clean and chemically stable MOP nanofilms that have high permeability and selectivity are still a challenge to fabricate.

All-carbon-structural and high-molecular-weight poly-*p*-xylylenes (Parylene), in which benzene rings are connected by ethylene linkers, have been widely used as protective coatings of printed circuits and medical devices, barrier layers of metal surfaces to prevent corrosion, and lubricants^17^. This polymer can be prepared by using a chemical vapor deposition (CVD) process of low-molecular-weight [2.2]paracyclophanes^18^. In this process, two *p*-xylylene monomers are formed by vacuum vapor pyrolysis of [2.2]paracyclophane above 550 °C, and the reactive monomers polymerize into pure poly-*p*-xylylenes on the surface of targets at room temperature in a vacuum ambient. This self-initiated process enables uniform poly-*p*-xylylene films to form on the target surfaces without voids or pinholes. Conformal coating of various devices with poly-*p*-xylylene films exhibits outstanding barrier properties for gasses, water, and organic solvents owing to dense packing of rigid polymer chains. If well-defined pore structures can be created in poly-*p*-xylylene nanofilms fabricated by the CVD dry process, microporous nanofilms are potentially useful materials for reliable and clean membranes that have selective separation functions.

Here, we report a new versatile approach to fabricate microporous nanofilms on the basis of poly-*p*-xylylenes without using wet processes. We used the CVD process with [2.2]paracyclophane derivatives to prepare uniform crosslinked poly-*p*-xylylene nanofilms less than 50 nm thick on ultrafiltration (UF) supports^19^. The polymerization of [2.2]paracyclophanes that have polar substituents can create interconnected hydrophilic micropores within the nanofilms. We measured the permeance of water and alcohols as well as the retention of various dye molecules. The crosslinked network of poly-*p*-xylylenes diminishes the formation of crystalline domains among rigid polymers, and enables the rapid permeance for organic solvents by enhancing of microporosity. In addition, the nanofilms exhibit an excellent separation performance for solutes.

## Deposition of Poly-*p*-xylylene Nanofilms by CVD

**Figure 1:**
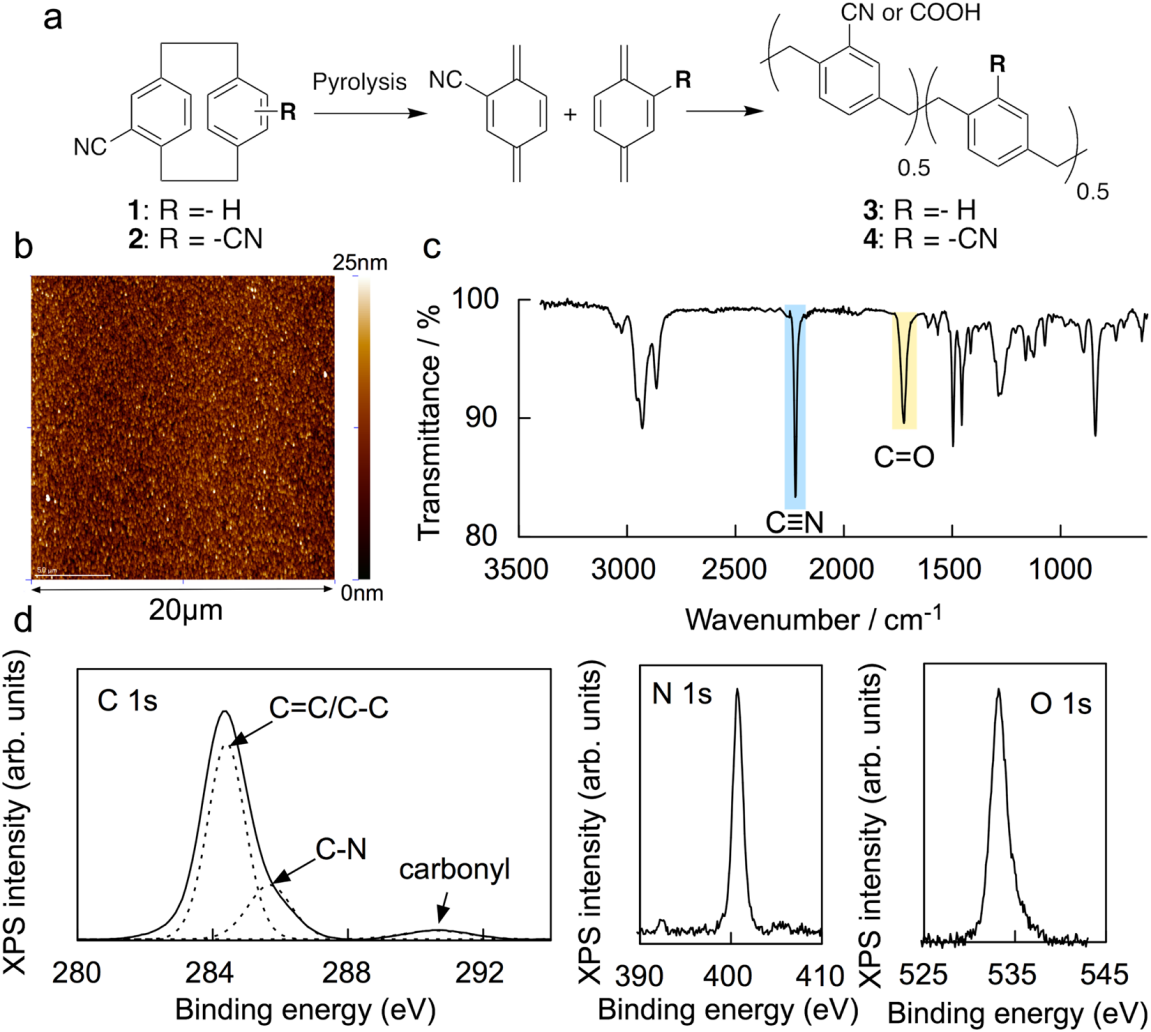
(**a**) Synthesis of poly-*p*-xylylenes **3** and **4** by CVD polymerization of **1** and **2**. (**b**) AFM image of **4** on Si wafer. (**c**) FT-IR spectrum of **4** deposited on a Si wafer. (**d**) C 1 s, N 1 s, and O 1 s XPS spectra for **4**. Raw data spectrum for C 1 s (solid line) is fitted with three components (dotted lines).

Since polar nitrile groups can form hydrogen bonds with water molecules, we designed and synthesized two [2.2]paracyclophanes **1** and **2** that have one or two nitriles (Figure 1a)^20^. Synthesized molecules **1** and **2** were sublimed by heating at 175 °C under vacuum pressure. Subliminated **1** and **2** were pyrolyzed to create monomer gas, and the formed monomers were polymerized into poly-*p*-xylylenes **3** and **4** at the surface of a Si wafer at room temperature. Films **3** and **4** did not dissolve in aqueous solutions (10% concentrations of HCl, H_2_SO_4_, NaOH, and NH_4_OH) or organic solvents (isopropyl alcohol, acetone, toluene, and chlorobenzene) at room temperature. The film thickness deposited on Si wafers is proportional to the initial amount of **1** or **2** (Fig. S1). Figure 1b shows an atomic force microcopy (AFM) image for the surface of **4** on a Si wafer. Poly-*p*-xylylene films **3** and **4** exhibited smooth and uniform surfaces composed of nanometer-sized particle assemblies that had height variations within 1.2 nm. The free volume sizes within **3** and **4** were detected by using positron (*e* ^+^) annihilation lifetime spectroscopy (PALS) (Fig. S2)^21,22^. The lifetime of ortho-positronium (*o*-Ps), which is generated by exposing an insulating material to a positron source ^22^Na, correlates with the average free volume size in materials. The *o*-Ps lifetimes of 1.74 and 1.66 ns for **3** and **4** correspond to the average diameters of 0.52 and 0.50 nm. These suggest that the CVD polymerization of **1** and **2** can create free volumes less than 1 nm in diameter within poly-*p*-xylylene nanofilms.

The chemical compositions of the poly-*p*-xylylene films **3** and **4** were characterized by Fourier transform infrared spectroscopy (FT-IR) and X-ray photoelectron spectroscopy (XPS) analyses. The FT-IR spectrum of **4** on the Si wafer showed two characteristic bands at 2220 and 1730 cm^−1^ assigned to C≡N and C = O stretching vibrations (Fig. 1c), whereas no C = O vibration band was not detected for the corresponding [2.2]paracyclophane **2**. Figure 1d shows the XPS spectrum for the C 1 s, N 1 s, and O 1 s of **4**. The spectrum consisted of three components with binding energies of 284.4 eV for C = C/C-C, 286.1 eV for C-N, and 290.8 eV for carboxyl groups^23^. Furthermore, **4** showed a clear O 1 s peak at 532.9 eV, which can be ascribed to oxygen bound to carbon. The hydrophobicity of the poly-*p*-xylylene film surface was evaluated by using the contact angles of a water droplet for **3** and **4** deposited on the Si wafers. The contact angles of **3** (87.2 ± 2.2°) and **4** (83.3 ± 1.5°) were less than 90°, and **4** has a lower contact angle than **3** (Fig. S3 **)**. From these results, nitrile groups in **1** or **2** were partially oxidized into carboxylic acid during vacuum vapor pyrolysis process, and the films **3** and **4** exhibit hydrophilicity because of the introduction of polar nitrile and carboxylic acid as a substituent of poly-*p*-xylylenes.

The wide-angle X-ray scattering (XRD) pattern of **3** showed a peak at 2*θ* = 19.5° with a shoulder peak at 2*θ = *17.0° (Fig. S4). These two peaks at 2*θ = *17.0 and 19.5° can be assigned to a monoclinic crystal structure (α phase) in the (020) plane and a hexagonal crystal structure (β phase) in the (400) plane, respectively^24^. The other film **4** displayed a single reflection peak at 2*θ = *22.4°. Both films **3** and **4** were a semi-crystalline material containing crystalline domains made from the packing of rigid polymer chains. The packing of polymer chains in **3** and **4** is anticipated to obstruct the permeability of small molecules through poly-*p*-xylylene nanofilms.

**Figure 2:**
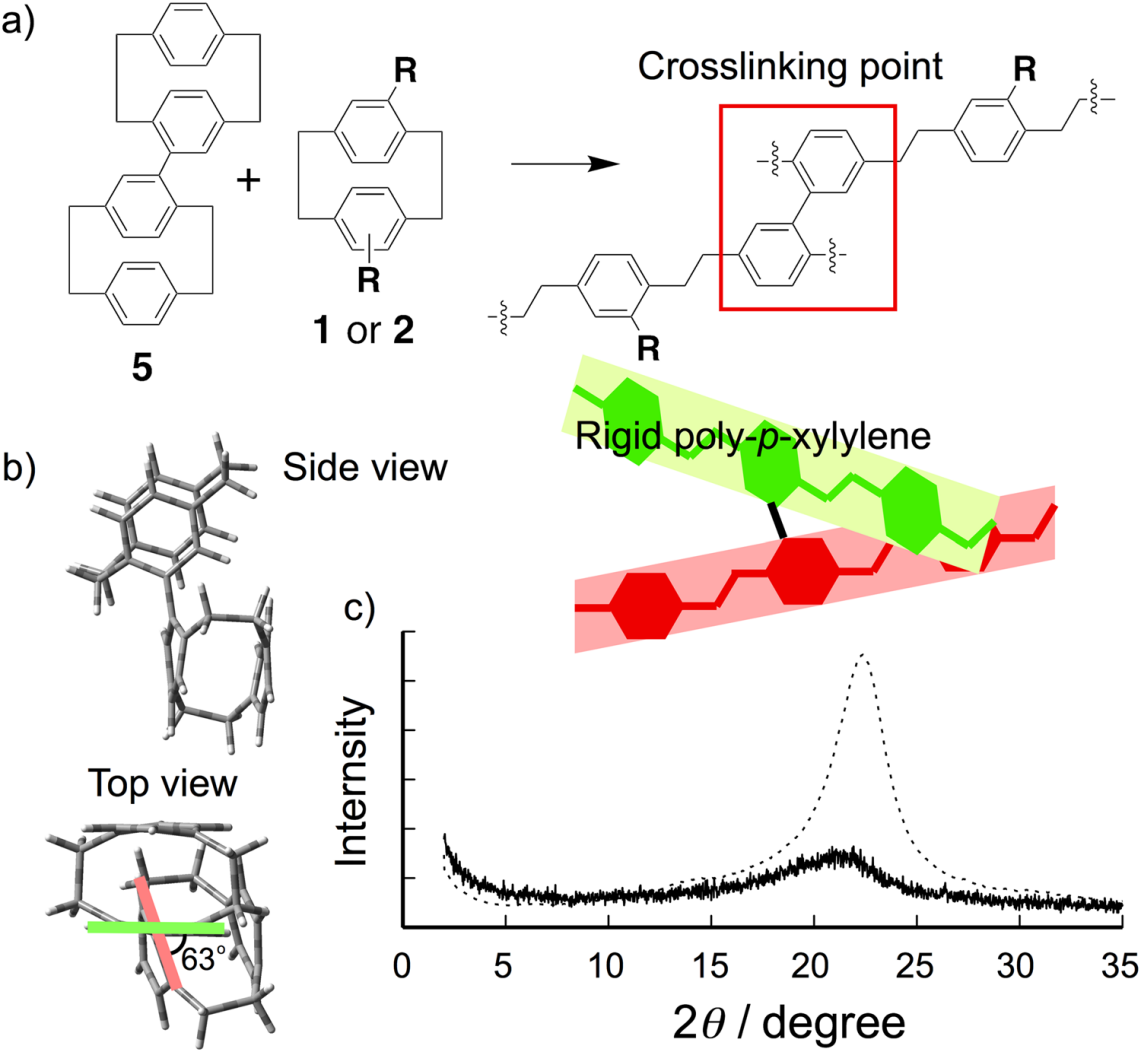
(**a**) Synthesis of crosslinked poly-*p*-xylylenes **6** and **7** by CVD polymerization **5** and **1** or **2**. (**b**) Molecular model of **5**. (**c**) XRD patterns reflected from poly-*p*-xylylene film **4** (dotted line) and crosslinked film **7** (solid line).

To prevent the packing of poly-*p*-xylylene chains, we designed the crosslinked polymer network by using a [2.2]paracyclophane dimer **5** (Fig. 2a). Dimer **5** was synthesized by the homo-coupling reaction of 4-bromo[2.2]paracyclophane in the presence of bis(1,5-cyclooctadiene)nickel(0) (Ni(cod)_2_) in 76% yield. In accordance with the simulated optimized structure of **5**, two [2.2]paracyclophane units in **5** were connected with a twisted biphenyl bridge, and the torsional angle between two units is estimated to be 63° (Fig. 2b). Films **6** and **7** deposited onto the Si wafer were prepared by polymerizing mixed powders of **5** with **1** or **2** under the same CVD conditions as **3** and **4**. Whereas the chemical compositions and the surface morphologies of **6** and **7** were mostly similar to those of **3** and **4** (Fig. S5), the XRD patterns of **6** and **7** revealed only a broad harrow around 2*θ* = 20°, suggesting the amorphousization of poly-*p*-xylylene films caused by the mixing of **5** (Fig. 2c and Fig. S4). The Vickers hardness (HV) values of a 50-nm-thick nanofilms **6** and **7** were significantly higher than those of **3** and **4** as evaluated by using a dynamic ultra microhardness tester with Vickers indenter (Table 1)^25^. The networking of poly-*p*-xylylenes by biphenyl bridge restricts the structural relaxation of polymer chains in the nanofilms. When the twisted biphenyl monomer from **5** reacts with the other monomer gases generated by the pyrolysis, these monomer gases can polymerize to form the crosslinked polymer network onto the target surface. The XRD and HV analyses of **3** and **4** suggested an inefficient packing of polymer chains as well as the enhancement of the robustness of the poly-*p*-xylylene nanofilms by the introduction of crosslinking points.

**Table 1:** Properties and filtration performance of poly-*p*-xylylene nanofilms 3, 4, 6, and 7.

Nanofilm	Monomer	Contact angle^a^ (deg)	HV^b^ (−)	Thickness (nm)	Water permeance^c^ (L m^−2^ hr^−1^ MPa^−1^)	Dye rejection ratio (%)^c^	
						RB^d^	MO^e^
**3**	**1**	87.2 ± 2.2	400 ± 175	41 ± 3	1.3 ± 0.3	>99	76 ± 2
**4**	**2**	83.3 ± 1.5	170 ± 15	43 ± 4	5.2 ± 0.8	>99	58 ± 2
**6**	**1 + 5**	84.5 ± 1.2	950 ± 100	40 ± 3	11.3 ± 2.0	>99	77 ± 3
**7**	**2** + **5**	82.0 ± 1.1	1150 ± 250	41 ± 3	30.5 ± 2.8	>99	87 ± 4
				24 ± 2	71.0 ± 9.0	>99	84 ± 3

^a^Contact angles were determined as the average values from 18 measurements for three nanofilms deposited on Si wafer (size: 2 × 2 cm). ^b^Average HV values were calculated as the average values from 12 measurements for two 50-nm-thick nanofilms deposited on Si wafer. The flatness of nanofilms on Si wafer was evaluated by AFM analyses. RMS roughness (*R*_q_) value for **3**: R_q_ = 4.5 nm; **4**: R_q_ = 4.0 nm; **6**: R_q_ = 3.5 nm; **7**: R_q_ = 3.2 nm. ^c^Data on water permeance and dye rejection performance was the average of five independent readings. ^d^RB: Rose Bengal. ^e^MO: Methyl Orange.

## Selective Solvent Permeance of Poly-*p*-xylylene Nanofilms

**Figure 3:**
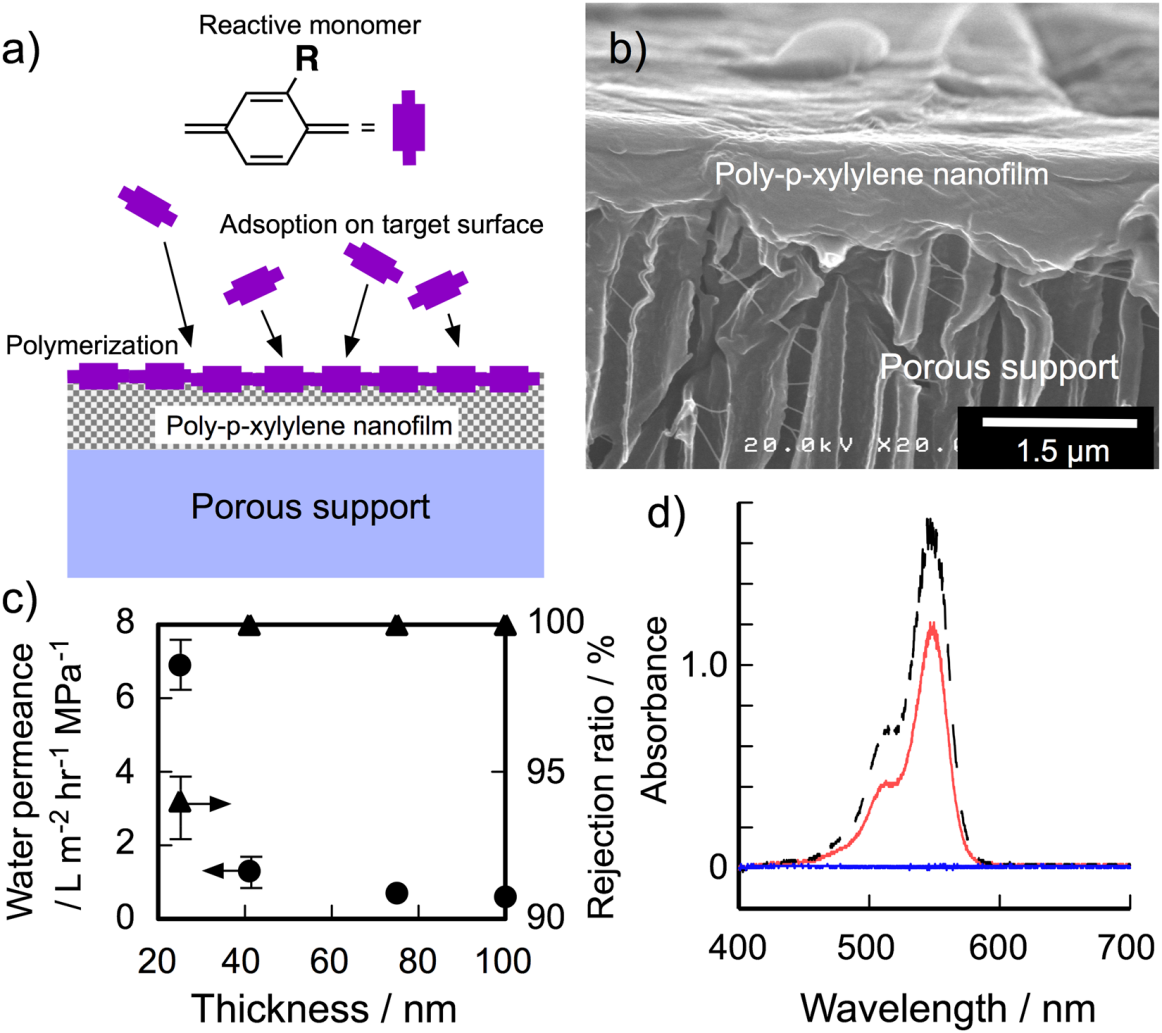
(**a**) Schematic representation of CVD polymerization onto porous supports. (**b**) FE-SEM image of surface of 41-nm-thick **4** film on a supporting membrane. (**c**) Dependence of water flux and dye rejection ratio ([Rose Bengal] = 10 μM) on film thickness of **3** by using dead-end membrane filtration system at a pressure difference of 0.5 MPa at room temperature. (**d**) UV-Vis spectra of Rose Bengal aqueous solutions ([dye] = 10 μM, red line) concentrated by (black dotted line) and permeated through (blue line) membrane coated with **4**.

Nanofilms were deposited onto a top dense layer of a UF membrane (Biomax polyethersulfone (normal molecular weight limit: 50 kDa)) to measure permeability for water and organic solvents (Fig. 3a). The scanning electron microscopy (SEM) image of the top of a UF membrane coated with **3** displays a smooth flat surface, indicating uniform covering of the supporting membrane surface with the poly-*p*-xylylene nanofilms (Fig. 3b and Fig. S6). The permeation of water was investigated for five membranes coated with **3**, **4**, **6**, **7** and poly-di-chloro-*p*-xylylene (Parylene C) by using the dead-end membrane filtration system. No permeation of water could be detected for the UF membrane with hydrophobic Parylene C nanofilm over a period of one week at a pressure difference of 0.5 MPa. In contrast, water permeation of a membrane with 41-nm-thick nanofilm **3** could be readily observed at the same pressure difference, and the average water permeance was 1.3 ± 0.3 L m^−2^ hr^−1^ MPa^−1^ (Table 1). The **3**-coated membrane showed an inverse proportionality between water permeance and film thickness (Fig. 3c and Fig. S7)^26^, and water permeance increased linearly with increasing pressure difference in the range of 0.1–0.5 MPa (Fig. S8). Nanofilm **4** made from **2** that had two nitriles had a permeance around four times higher than that of **3**, suggesting that pores within **4** are more hydrophilic than those in **3** due to the high density of polar substituents in poly-*p*-xylylenes. Water could permeate through water-permeable domains in the poly-*p*-xylylene nanofilms **3** and **4** driven by the pressure difference.

To evaluate the filtration performance of the hydrophilic poly-*p*-xylylene nanofilms, four water-soluble dyes that had different molecular sizes and charges were used as probes^27^. The rejection ratios (*R*) of four dyes were determined in accordance with the equation (C_f_-C_p_)/C_f_ × 100, where C_f_ and C_p_ are the dye concentrations of the feed and permeate sides of the dead-end membrane filtration system (Table 1). The membrane with **3** demonstrated almost 100% rejection of negative-charged Rose Bengal (1.13 nm × 1.20 nm, Fig. S9) as shown in Fig. 3d, indicating that the nanofilms contained no voids or pinholes. It was observed that positive-charged Rhodamine 6 G and negative-charged Methylene Blue were retained almost completely by the membrane, indicating that the rejection does not depend on the charges of dyes. From the rejection ratios for dyes, the free volumes of the poly-*p*-xylylene films were calculated using the Ferry-Renkin equation *R* = [1–2(1 − *a*/*r*)^2^ + (1 − *a*/*r*)^4^] × 100, where *R* is the rejection ratio, *a* is the radius of the dye, and *r* is the radius of the free volume^28^. The *r* for **3** and **4** were estimated to be less than 0.6 nm. These *r* values were almost double of the free volume sizes for dried samples determined by PALS, suggesting the enlargement of free volume size by filling with water. The molecular weight cut-off (MWCO) for **3** and **4** were 1500 g/mol, determined from the rejection measurements of polyethylene glycols with different molecular weights (Fig. S10). From these results, poly-*p*-xylylene nanofilms **3** and **4** work as selective layers that have a molecular size cutoff in accordance with their free volume size.

The membranes coated with crosslinked nanofilms **6** and **7** showed higher water permeance values than non-crosslinked **3** and **4**, indicating the enhancement of microporosity created by the inefficient packing of polymer chains within the crosslinked nanofilms. Although decreasing the thickness of **3** resulted in lowering of the Rose Bengal rejection as shown in Fig. 3c, water permeance of 24-nm-thick **7** is as high as 71.0 ± 9.0 L m^−2^ hr^−1^ MPa^−1^ while maintaining the complete rejection of Rose Bengal (Table 1). The rejection ratio of small Methyl Orange was improved by the networking of polymer chains (Fig. S11). This can be attributed to the fluctuation of polymer chains being suppressed in the crosslinked polymer networks, which is also supported by the enhanced film stiffness. Membranes comprising crosslinked poly-*p*-xylylene nanofilms fabricated by the CVD polymerization of two monomers show outstanding separation performance in water with water permeance of the same level of water permeance as those of commercial NF membranes (30–80 L m^−2^ hr^−1^ MPa^−1^)^21,29^.

**Figure 4:**
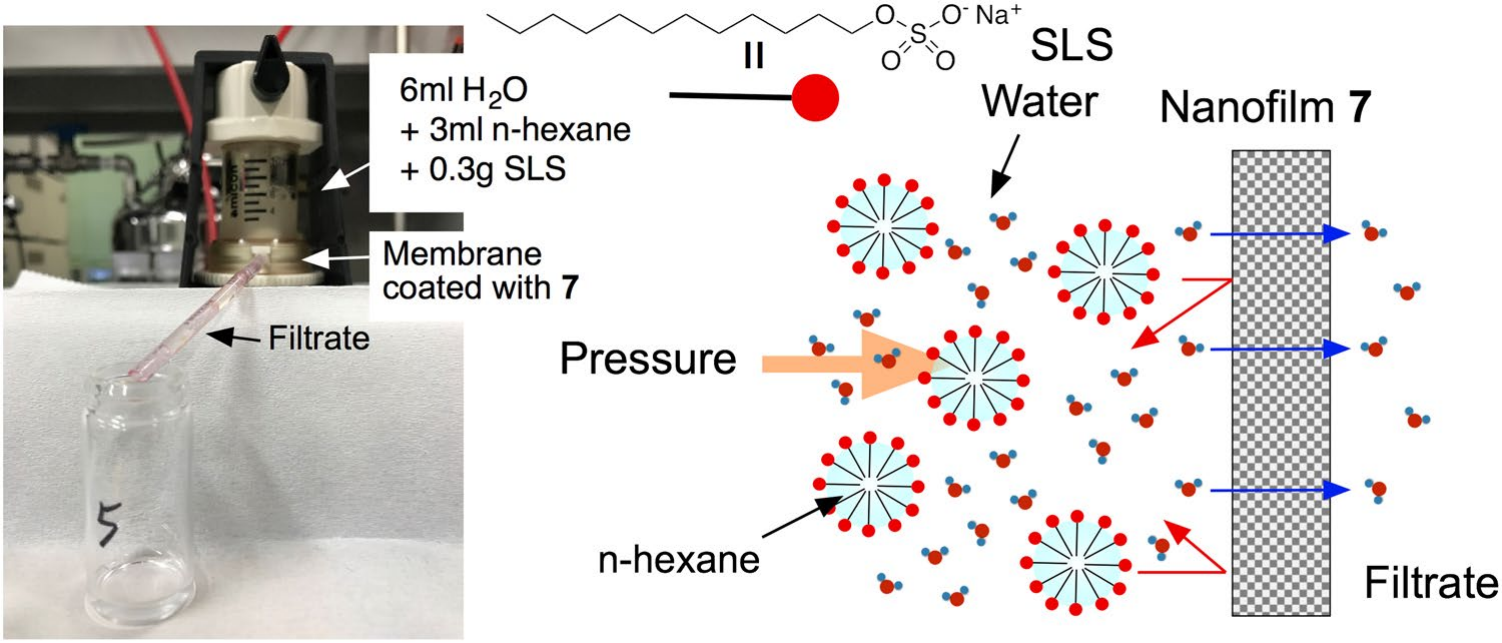
Permeation equipment for mixed solution of water and n-hexane in the presence of SLS. Schematic representation of selective protection of n-hexane and SLS from aqueous dispersion.

We also investigated the permeation properties of organic solvents (n-hexane, methanol, ethanol, and 2-propanol) for the membranes coated with a 41-nm-thick nanofilm **7**. Use of solvent-resistant nanofiltation membranes for non-aqueous mediums holds strong potential for food, refining, and pharmaceutical industries^30^. Methanol achieved the highest permeance of 54.5 ± 5.0 L m^−2^ hr^−1^ MPa^−1^, and the permeance of alcohols decreased as the molecular size increased (ethanol: 42.0 ± 6.0 L m^−2^ hr^−1^ MPa^−1^; 2-propanol: 24.0 ± 3.0 L m^−2^ hr^−1^ MPa^−1^)^5^. On the other hand, n-hexane did not pass through this membrane, suggesting that the nanofilm **7** rejected the penetration of non-polar n-hexane. When n-hexane was dispersed into water as an emulsion by using sodium lauryl sulfate (SLS), the filtrate that passed through the membrane coated with **7** did not contain n-hexane and SLS as monitored by FT-IR (Fig. S12 **)**. The nanofilm **7** can separate water from the mixtures of alkanes and water in the presence of surfactants (Fig. 4 **)**.

## Discussion

We have demonstrated the deposition of defect-free MOP nanofilms onto UF membranes fabricated by a dry CVD polymerization process of [2.2]paracyclophanes that have polar substituents. Although the ultrathin nanofilms worked as selective layers for aqueous solution that had a sharp molecular size cutoff in accordance with their free volume sizes, their water permeance was much lower than those of previously reported membranes^21,29^ and the dye rejection ratio was decreased by reducing of the film thickness. These were mainly due to the packing of linear polymer chains and the fluctuation of polymer chains. To achieve high water permeance while keeping selectivity high, we introduced the crosslinking points within poly-*p*-xylylene nanofilms by using twisted [2.2]paracyclophane dimer **5**. The introduction of twisted crosslinking points into poly-*p*-xylylenes led to enhanced microporosity as well as improved mechanical strength. The membranes coated with thinner crosslinked MOP nanofilms offer outstanding performance in terms of nanofiltration in water and alcohols. Moreover, we found the blocking property of non-polar organic solvent permeation through the nanofilms, suggesting the specific interaction of hydrophilic pores with organic solvents. The nanofilms efficiently recovered water from an oil-water emulsion. Efficient membrane separation of oil and water can contribute to minimamize chemical processes in oil recovery and water purification^31^. Since free volume size can be tuned by modifying substituents in [2.2]paracyclophane, robust and clean nanofilms fabricated by the CVD process have a great potential to realize efficiently separate membranes for various target molecules.

## Methods

### General

Surface morphologies of nanofilms were characterized using atomic force microscopy (AFM) images in non-contact mode by a JEOL JSPM-5400 system and field-emission scanning electron microscope (FE-SEM) images recorded in a Hitachi S-5000 FE-SEM with Pt sputter coating. IR spectra were obtained on a Shimazu IR Prestige-21 with DuraSample IR II. The crystalline structure of the samples was analyzed using wide-angle X-ray diffraction (Rigaku XRD-DSC) with Cu Kα radiation. The chemical compositions of nanofilms were analyzed using X-ray photoelectron spectroscopy (Kratos Analytical Axis Ultra). The free volume sizes of dry nanofilms **3** and **4** were analyzed by positron annihilation lifetime spectroscopy system (Fuji Imvac. PALS-200A). The pore size in the nanofilms was obtained from the annihilation lifetime-positron count curve according to the reported method^32^. The Vickers hardness (HV) values of a 50 nm-thick deposited on Si wafers were determined by dynamic ultra microhardness tester (Shimazu DUH-211) with Vickers indenter.

### Syntheses of 1, 2, and 5

**1**: 4-Bromo[2.2]paracyclophane was synthesized by standard iron-catalyzed bromination of [2.2]cyclophane in 76% yield^33^. The mixture of 4-bromo[2.2]paracyclophane (0.25 g, 0.87 mmol) and CuCN (0.10 g, 1.10 mmol) in 10 ml 1-methyl-2-pyrrolidone (NMP) was heated at 220 °C with stirring for 20 h under N_2_. After cooling, the reaction mixture was poured into 10 ml of 25 wt% aqueous ammonia solution and stirred for one night. The solid in the mixture was dissolved in CH_2_Cl_2_ and the organic layer was washed with 20 ml water for three times. The organic layer was dried with MgSO_4_ and evaporated. The product was purified by column chromatography (silica gel, eluent; CH_2_Cl_2_:n-hexane = 1:1 v/v) and sublimination to give a white solid. Yield 0.10 g (50%). ^1^H-NMR (CDCl_3_, 400.13 MHz): *δ* = 6.90 (d, *J* = 8.4 Hz, 1 H, Ar***H***), 6.75 (s, 1 H, Ar***H***), 6.52 (dd, *J* = 8.0 Hz, 1 H, Ar***H***), 6.57 (d, *J* = 8.0 Hz, 1 H, Ar***H***), 6.52 (t, *J* = 1.2 Hz, 2 H, Ar***H***), 6.48 (d, *J* = 7.6 Hz, 1 H, Ar***H***), 3.54–3.47 (m, 1 H, -C***H***_***2***_-), 3.32–3.26 (m, 1 H, -C***H***_***2***_-), 3.16–2.99 (m, 6 H, -C***H***_***2***_-). ^13^C-NMR (CDCl_3_, 100.61 MHz): *δ* = 143.2, 139.9, 138.5, 138.0, 136.1, 135.7, 133.5, 132.5, 131.8, 131.6, 129.9, 117.9, 113.8, 34.3, 34.0, 33.4, 33.2. FT-IR: 2220 cm^−1^ (-CN). ESI-TOF HRMS (APCI): *m/z* 234.1215 (M + H), calcd. for C_17_H_15_N: *m/z* 233.1204.

**2** was synthesized from 4, 4′-dibromo[2.2]paracyclophane by the literature method^34^. Yield 47%. ^1^H-NMR (CDCl_3_, 400.13 MHz): *δ* = 7.05 (d, J = 8.0 Hz, 2 H, Ar***H***), 6.81 (d, J = 2.0 Hz, 2 H, Ar***H***), 6.75 (dd, J = 2.0 Hz, 8.0 Hz, 2 H, Ar***H***), 3.60–3.53 (m, 2 H, -C***H***_2_-), 3.34–3.06 (m, 6 H, -C***H***_2_-). ^13^C-NMR (CDCl_3_, 100.61 MHz): δ = 144.2, 141.4, 137.0, 132.7, 118.8, 116.1, 35.2, 33.4. FT-IR: 2218 cm^−1^ (-CN). ESI-TOF HRMS (APCI): *m/z* 259.1150 (M + H), calcd. for C_18_H_14_N_2_: *m/z* 258.1157.

**5** ^35^: The mixture of 4-bromo[2.2]paracyclophane (1.74 g, 3.48 mmol), Ni(cod)_2_ (2.00 g, 4.18 mmol), 1,5-cyclooctadiene (1.01 ml, 4.18 mmol), and 2,2′-bipyridyl (1.14 g, 4.18 mmol) in 180 ml dry THF was heated at 60 °C with stirring for 40 h under N_2_. After cooled down to room temperature, the mixture was extracted with EtOAc, washed with brine, dried over with MgSO_4_, and concentrated under reduced pressure. The crude product was purified by column chromatography (silica gel, eluent: CH_2_Cl_2_:n-hexane = 1:1 v/v; R_f_ = 0.63) to afford **5** as a white powder. Yield 2.63 g (76%). ^1^H-NMR (CDCl_3_, 400.13 MHz): *δ* = 6.42–6.64 (m, 12 H, Ar***H***), 6.27–6.36 (m, 2 H, Ar***H***), 2.68–3.46 (m, 16 H, -C***H***_***2***_-). ^13^C-NMR (CDCl_3_, 100.61 MHz): *δ* = 140.4, 140.1, 139.9, 139.7, 139.5, 139.0, 138.2, 136.8, 135.5, 134.1, 133.3, 133.2, 133.0, 132.9, 132.8, 132.5, 132.2, 132.1, 130.9, 130.1, 36.0, 35.9, 35.3, 35.0. ESI-TOF HRMS (APCI): *m/z* 415.2450 (M + H), calcd. for C_32_H_30_: *m/z* 414.2348.

#### CVD process for poly-*p*-xylylene nanofilms

Poly-*p*-xylylene nanofilms were deposited onto UF membranes by using a Parylene deposition equipment (Specialty Coating System Inc., PDS 2010 Labcoter). [2.2]Paracyclophanes **1** and **2** were sublimed at 175 °C under vacuum pressure and pyrolyzed at 650 °C to create diradical reactive intermediate. The gaseous intermediate was then fed into a deposition chamber kept at room temperature and the intermediate could efficiently polymerize on the surfaces of UF membranes (Biomax polyethersulfone (normal molecular weight limit: 50 kDa, diameter: 25 mm)) and Si wafer to form nanofilms **3** and **4**. Crosslinked nanofilms **6** and **7** were prepared from the mixed powders of **1** or **2** with **5** (**1** or **2**: **5** = 2: 1 w/w) under the same CVD condition of **3** and **4**. The film thicknesses of nanofilms on Si wafer were determined by using a surface profiler (Kosaka laboratory Ltd., ET4000).

#### Nanofiltration

Nanofiltration experiments were carried out in repeats of five in a dead-end stirred cell (Merck Millipore, Model 8010) at 20 °C and 0.5 MPa. Data on water permeance and dye rejection performance was the average value by using five membranes coated with nanofilms, which were fabricated by five independent processes under the same conditions. The measurements were done after initial compaction phase until a permeance reached a steady state. The effective membrane area was 4.1 cm^2^ and stirring speed was 500 r.p.m. Permeate sample for solvent permeance measurements were collected intervals of 10 min and samples for rejection evaluations were taken after 2 hr. The solvent permeance (*J*, L m^−2^ hr^−1^ MPa^−1^) was determined by the equation *J* = *V*/(*A* x *t* x *p*), where *V* is the permeate volume, *A* is the effective membrane area, *t* is the unit time, and *p* is the applied pressure. The solute rejection tests were carried out using solutions ([dye] = 10 μM) containing one of the following dyes in water: Rose Bengal (1017 g/mol); Rhodamine 6 G (479 g/mol); Methylene Blue (319 g/mol); and Methyl Orange (327 g/mol) (Fig. S9). Dye concentrations of solutions were determined from the absorption intensities at the absorption maximum of each dyes. The MWCO values were determined from the rejection tests of polyethylene glycols in permeate samples by gel permeation chromatography^36^. The MWCO value was the molecular weight of polyethylene glycol with a rejection of 90%. A feed solution consisted of a mixture of polyethylene glycols with different molecular weights ranging from 330 to 8600 g/mol in a concentration of 1 g/L.

## Electronic supplementary material

Supporting Information
