# Supplementary material for: Selective Blocking Property of Microporous Polymer Membranes Fabricated by Chemical Vapor Deposition
Source: Sci Rep. 2017 Nov 15;7:15596. doi: 10.1038/s41598-017-15470-x (PMC5688126; doi:10.1038/s41598-017-15470-x)
Supplement: Supplementary file 1 — Supporting Information [file 41598_2017_15470_MOESM1_ESM.pdf]

Selective Blocking Property of Microporous Polymer Membranes Fabricated by Chemical Vapor Deposition

Takeshi Shii[1], Masaru Hatori[1], Kazuma Yokota[1], Yoshiyuki Hattori[1], and Mutsumi Kimura[1,2]*

[1]*Department of Chemistry and Materials, Faculty of Textile Science and Technology, Shinshu University, Ueda 386-8567, Japan*

And

[2]*Global Aqua Innovation Center, Shinshu University, Nagano 380-8553, Japan*

Supporting Information

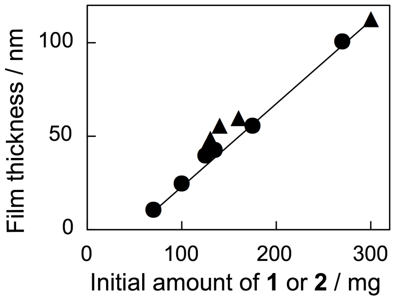

Fig. S1. Dependence of film thickness of **3** (●) and **4** (▲) on the initial amount of **1** or **2**.

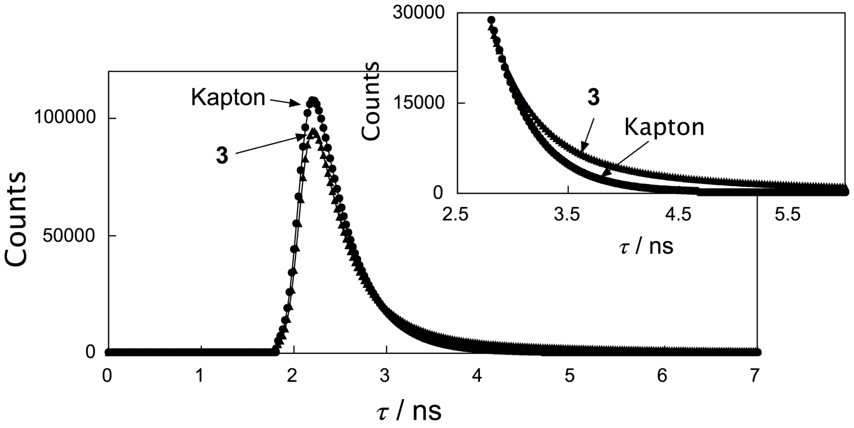

Fig. S2. Normalized positron annihilation lifetime spectra of poly-*p*-xylylene nanofilm **3** and Kapton film as a reference. *o*–Ps lifetime were obtained from the slope of decay curve as shown in the inset.

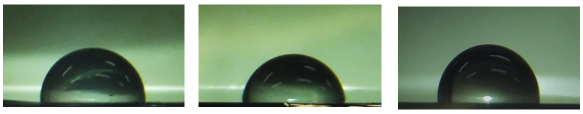

3 **4** Parylene C

Fig. S3. Water contact angles for **3**, **4** and Parylene C deposited on Si wafers.

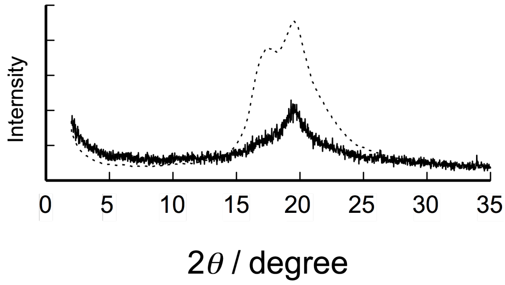

Fig. S4. XRD patterns reflected from poly-*p*-xylylene film **3** (dotted line) and crosslinked film **6**.

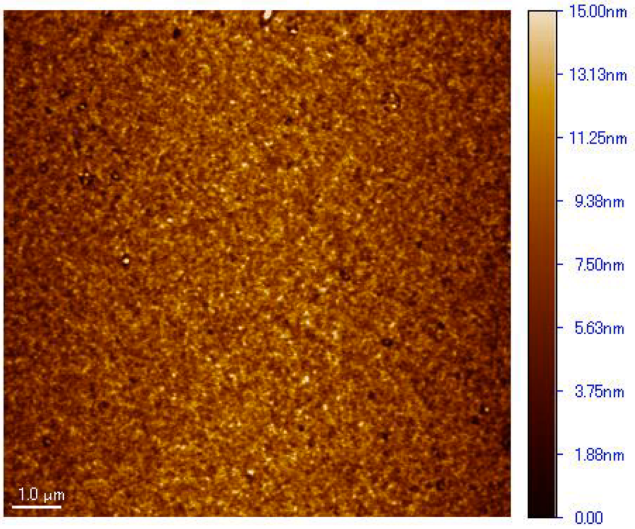

Fig. S5. AFM image of crosslinked poly-*p*-xylylene film **7** on a Si wafer.

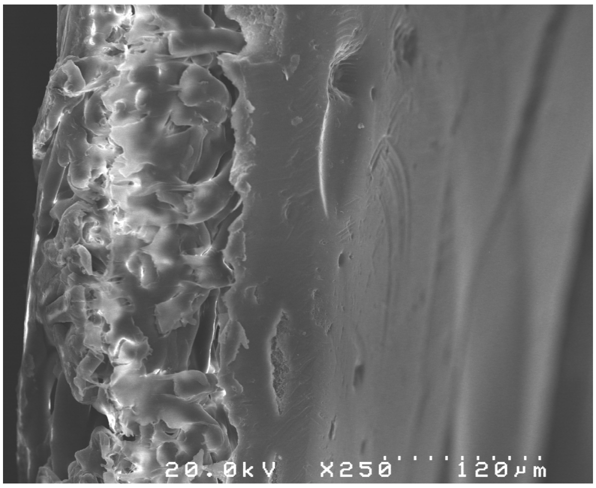

Fig. S6. Cross-cut FE-SEM image of UF membrane coated with crosslinked poly-*p*-xylylene nanofilm **7**.

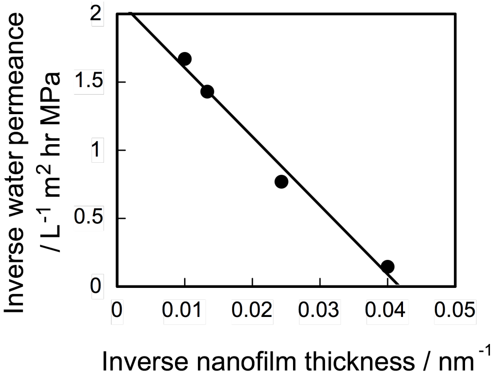

Fig. S7. Inverse of the water permeance of **3**-coated membrane as a function of the reciprocal nanofilm thickness.

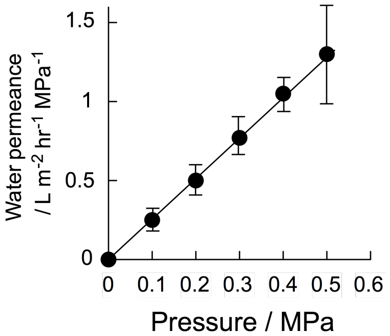

Fig. S8. Dependence of water permeance of **3**-coated membrane on the applied pressure.

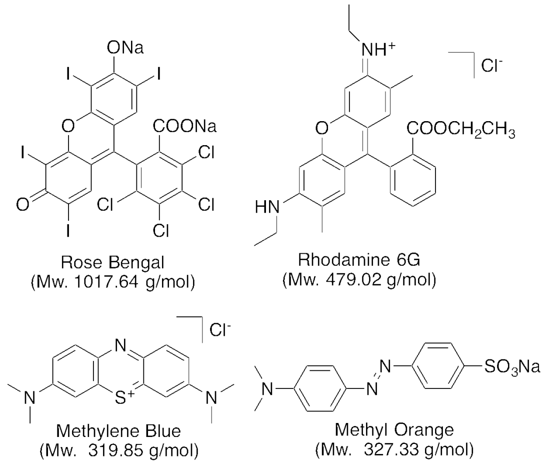

Fig. S9. Chemical structures of water-soluble dyes for the solute rejection tests.

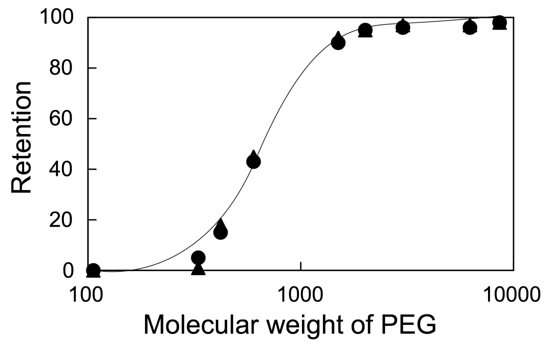

Fig. S10. Determination of MWCOs for **3** (●) and **4** (▲) from the rejection curve of PEGs.

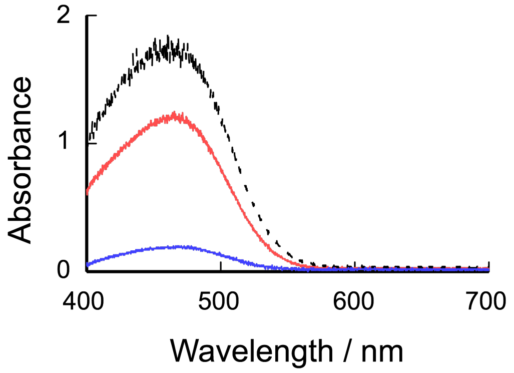

Fig. S11. UV-Vis spectra of Methyl Orange aqueous solutions ([dye] = 10 μM, red line) concentrated by (black dotted line) and permeated through (blue line) membrane coated with 24 nm-thick **7**.

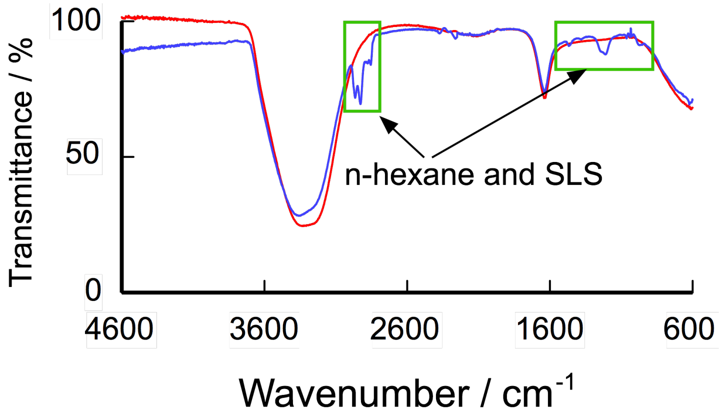

Fig. S12. FT-IR spectrum of filtrate (red line) permeated through membrane coated with 41 nm-thick **7**. Blue line is the FT-IR spectrum of emulsion solution of water-n-hexane in the presence of SLS.
